# Rare intestinal fistula caused by primary lymphoma of the gastrointestinal tract: Two case reports and literature review

**DOI:** 10.1097/MD.0000000000011407

**Published:** 2018-07-06

**Authors:** Nan Zhuang, Qingli Zhu, Wenbo Li, Miaoqian Wang, Qian Yang, Wei Liu, Ji Li, Hong Yang, Weixun Zhou

**Affiliations:** a Department of Ultrasound; b Department of Radiology; c Department of Gastroenterology; d Department of Pathology, Peking Union Medical College Hospital, Beijing, China.

**Keywords:** intestinal fistula, intestinal lymphoma, ultrasound

## Abstract

**Rationale:**

Primary lymphoma that arises from the intestine is an uncommon malignant tumour, while intestinal fistula caused by primary lymphoma is even rarer. Non-specific clinical performance makes early diagnosis difficult, although imaging modalities might play an essential role in the detection of intestinal fistula.

**Patient concerns:**

Patient 1: A 60-year-old male hospitalized with diarrhoea and abdominal pain for seven months underwent computed tomography enterography (CTE) that demonstrated ileum internal fistula and ileac-sigmoid colon fistula. Ultrasound (US) showed small intestinal wall thickened and development of a fistula of the sigmoid colon due to malignance. Patient 2: A 43-year-old male presented with abdominal pain and diarrhoea lasting one year. US revealed a fistula between the sigmoid colon and the ileum, and CTE showed that the wall of the partial sigmoid colon was abnormally thickened and enhanced with an ileal-sigmoid fistula that strongly suggested the diagnosis of lymphoma.

**Diagnoses:**

Both the two patients were diagnosed as intestinal fistula caused by primary non-Hodgkin's intestinal lymphoma.

**Interventions:**

The patient 1 underwent surgery followed by chemotherapy. The patient 2 accepted chemotherapy.

**Outcomes:**

Two patients’ general conditions remained stable and the imaging revealed no recurrence after follow-up of about 12 months.

**Lessions:**

Cross-sectional imaging, such as US and CT, plays an essential role in intestinal lymphoma fistula diagnosis.

## 1 Introduction

Primary lymphoma is a rare malignant tumor in the gastrointestinal (GI) tract that accounts for approximately 1% to 4% of all GI malignancies.^[1]^ The intestine is the second most commonly implicated site of occurrence after the stomach.^[2]^ A tumor bowel fistula is an uncommon but dangerous complication in the advanced stages of primary intestinal lymphoma. Because the clinical symptoms and signs are not specific, imaging modalities can help in detecting this complication. Few intestinal fistulas caused by primary lymphoma have been reported.^[3,4]^ In this report, we describe the clinical manifestation, imaging features, treatment, and prognosis of 2 cases of intestinal fistula that were complications of small intestine lymphoma.

## 2 Case report

### 2.1 Case 1

**Figure 1:**
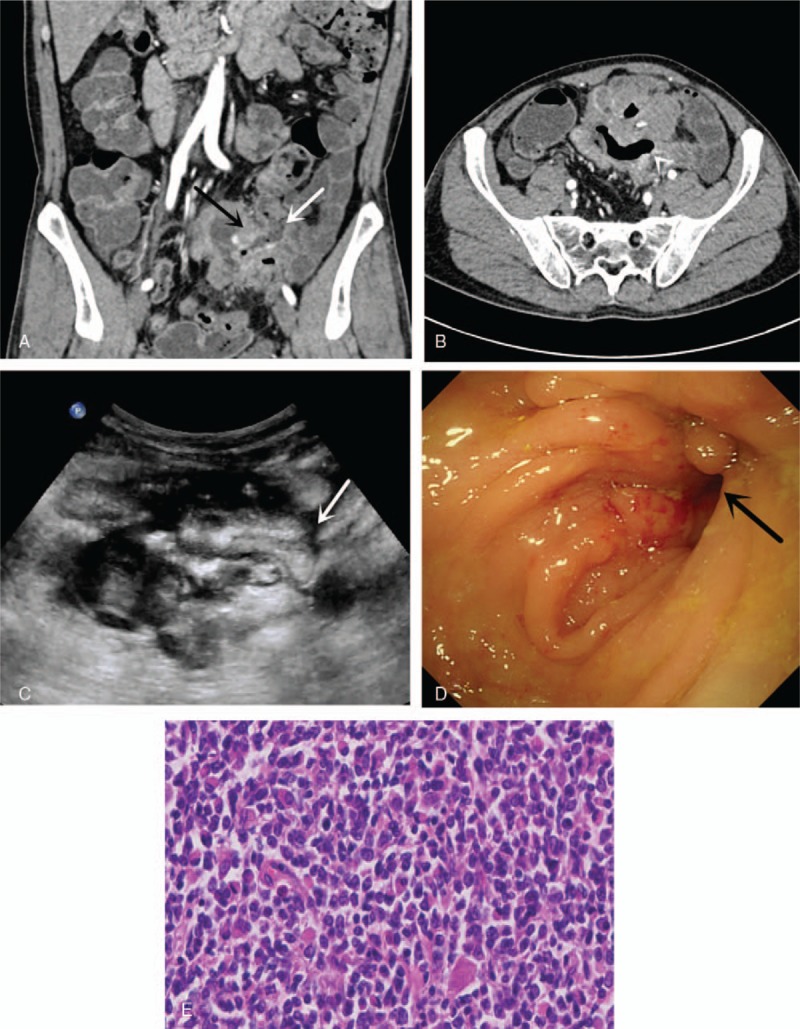
(A) Wall of ileocecal junction (black arrow), remarkably thickened and enhanced, that could not be separated from the sigmoid colon (white arrow). The lumen of the sigmoid colon is remarkably narrow. (B) A fistulous tract between the pelvic intestine and sigmoid colon. (C) Transabdominal US showed the remarkably uneven thickness and hypoecho terminal intestinal wall; the thickest part measured 1.9 cm. The ileum could not be separated from the sigmoid colon. Interruption of the continuity of the sigmoid colon wall can be seen (white arrow) where a fistula exists between the ileum and the sigmoid colon. (D) Enteroscopy showed a fistula (black arrow) in the sigmoid colon approximately 28 cm from the anus, whose adjacent mucosa was edematous and disordered. (E) Histopathological examination demonstrated primary non-Hodgkin's intestinal lymphoma (large diffuse B-cell lymphoma).

A 60-year-old male was hospitalized with the primary complaint of diarrhea and abdominal pain for over 7 months. He mainly presented a pinching pain around the umbilicus and watery diarrhea. On physical examination, body mass index (BMI) was 20.1 kg/m^2^, and an approximately 3-cm-diameter, relatively hard, slightly movable mass was palpable in the left lower abdomen without obvious tenderness or superficial lymphadenopathy. Laboratory examination showed the following positive findings: C-reactive protein level (CRP) was 12.14 mg/L↑ (normal: 0.1–10.0 mg/L) and fecal occult blood (OB) was positive (+). The blood routine, erythrocyte sedimentation rate (ESR), set of tumor markers, antinuclear antibody spectrum (ANAs), and inflammatory bowel disease antibody spectrum showed no abnormalities. Computed tomography enterography (CTE) demonstrated that the regional 6th small intestine wall was enhanced with multiple air pockets inside the involved bowel. The lesion abutting the ileocecal junction and sigmoid colon had a distorted contour (Fig. 1A and B). The ileum internal fistula and ileac-sigmoid colon fistula were highly suggestive of malignancy. Transabdominal ultrasound (US) was then performed rather than an enteroscopy. Abdominal US revealed remarkably uneven thickening of the small intestinal wall in the pelvic area. The serosa layer of involved intestines remained intact and smooth. The most thickened part measured 1.9 cm. Colour Doppler flow imaging (CDFI) demonstrated that the inferior mesentery artery was thickened and was wrapped by the involved small intestine. The sigmoid colon was inseparable from the involved small intestine. Several enlarged mesenteric lymph nodes could be seen around the lesion (Fig. 1C). The US imaging features also indicated that the thickened intestinal wall and the fistula developed as a result of the tumors. Photon emission tomography/computed tomography (PET/CT) suggested that lymphoma was a very likely diagnosis. The patient underwent enteroscopy under local anesthesia. The enteroscope was passed smoothly into the terminal ileum by approximately 15 cm and but was unable to be further inserted as a result of extreme pain. The enteroscopy showed that the mucosa of the terminal ileum was congestive and edematous with sporadic erosion. A fistula appeared in the sigmoid colon approximately 28 cm from the anus, and the adjacent mucosa was edematous and disordered (Fig. 1D). A biopsy was performed on the lesions in the terminal ileum and sigmoid colon. Unfortunately, the biopsy sample showed chronic inflammation in pathology and thus failed to provide a clear diagnosis. The patient chose to have an operation. The part of the terminal ileum near the cecum and the sigmoid colon was found to form an adhesion, accreting in the superficial region of the inferior mesenteric vessels during the operation. Then, the surgeon performed accretion lysis, right hemicolectomy, sigmoidectomy, and ileostomy. The ileocecal valve and parts of the colon were removed. Histopathological examination demonstrated a gray 6 × 5 × 3.5-cm nodule that was found in the serosa of the intestine 7 cm from the ileocecal valve. The mass was adhered to another portion of the intestine where a fistula could be seen. Three other gray masses were also found 15, 20, and 31 cm from the ileocecal valve. They could not be separated from the surrounding tissue. Histopathological examination proved the diagnosis of primary non-Hodgkin's intestinal lymphoma (large diffuse B-cell lymphoma) (Fig. 1E). Postoperatively, the patient received 8-course chemotherapy with R-CHOP. For nearly 12 months, his general condition remained stable, and intestinal imaging reexaminations showed no abnormalities after stoma closure.

### 2.2 Case 2

**Figure 2:**
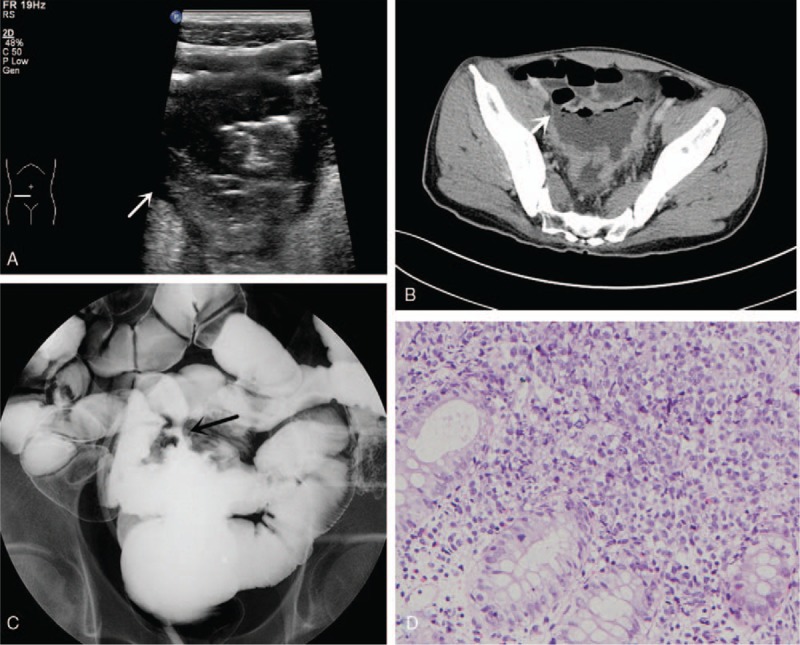
(A) US shows the sigmoid colon adhered to the pelvic small intestine, a fistulous communication (white arrow) in which intestinal content that is moving can be seen between them. (B) CTE demonstrating that the wall of the partial sigmoid colon was abnormally thickened and kept a close relationship with the small intestine, suggesting an intestinal fistula (white arrow). (C) Barium enema showed a tract (black arrow) between the small intestine and the sigmoid colon. (D) Histopathologic result showed non-Hodgkin's large diffuse B-cell lymphoma.

A 43-year-old male who presented with abdominal pain and diarrhea lasting 1 year was admitted to our hospital. He started presenting with hematochezia and lower fever 1 month before admission. On physical examination, his BMI was 17.58 kg/m^2^, and no mass could be distinctly palpated on his scaphoid abdomen. Laboratory examinations showed the following blood and biochemical findings: 95 g/L hemoglobin, OB (+) stool, and 20.24 mg/L CRP, negative for the entire set of tumor markers and negative for T-SPOT.TB (tuberculosis). The transabdominal US demonstrated that the intestinal wall of the sigmoid colon was irregularly thickened and had a loss of normal construction, presenting a hypoechoic mass as the rough serosa. Increased blood flow signal was also detected in the intestinal wall. The sigmoid colon was found adhered to the abutting pelvic small intestine. A fistulous communication was confirmed when intestinal content was moving between the sigmoid colon and the ileum during a real-time dynamic US scan (Fig. 2A). Multiple enlarged pelvic lymph nodes were nearby. Barium enema examination showed a tract between the small intestine and the sigmoid colon, where the wall was stiff, and the lumen was narrow. Contrast-enhanced CT and intestinal reconstruction demonstrated that the wall of the partial sigmoid colon was abnormally thickened and enhanced with an ileal-sigmoid fistula that strongly suggested the diagnosis of lymphoma (Fig. 2B and C). PET/CT showed an irregular hypermetabolic focus located between the rectum and the sigmoid (SUVmax: 16.0) that was suspected to be a malignant lesion. Enteroscopy revealed a large ulceration from the sigmoid-rectal junction to the segment 12 cm above the anus. One side of the ulceration formed a fistula, from which smooth intestinal mucosa could be seen. The sigmoid-ileum fistula was confirmed. The histopathologic result showed non-Hodgkin's large diffuse B-cell lymphoma (Fig. 2D). Because of the large lesion, severe adhesion and lack of surgical indication, the patient accepted chemotherapy with rituximab, cyclophosphamide, doxorubicin, vincristine (R-CHO), and prednisone (R-CHOP) after the first chemotherapy treatment rather than the operation.

The patient had sudden central abdominal colic pain with discontinued exhaust and defecation after eating oatmeal when 2 courses of chemotherapy were completed. Emergency CT suggested mechanical ileus. The patient improved with nonsurgical therapy. In the following treatment course, the patient maintained a relatively stable condition by continuous consumption of a liquid or semi-liquid diet. PET/CT showed remarkable shrinkage of the rectosigmoid lesion size and decreased metabolic activity after 4 courses of chemotherapy. The patient finished 8 courses of chemotherapy. During the 12-month follow-up period, colonoscopy and CTE did not reveal obvious fistula, and his diet and defecation were good.

## 3 Discussion

Spontaneous tumor bowel fistulas are generally reported to be caused by cervical, ovarian, and colon cancers.^[5]^ Primary lymphoma causing tumor-bowel fistula without chemotherapy, as reported in our cases, is rarely reported. A retrospective review^[6]^ of all GI lymphoma patients over a 37-year period found that 9% of the patients developed a perforation, 55% of which occurred after chemotherapy. The most common site of perforation was the small bowel (59%), followed by the large bowel (22%) and the gastric tract (16%).^[6]^ According to the literature, intestinal fistula caused by lymphoma could affect the adjacent loops, the bladder and even the aorta.^[7–9]^

Fistula-formation is a chronic and long-term process. In the early period of primary intestinal lymphoma, the tumors are confined in the submucosa; with the progression of the course of lymphoma, tumors gradually invade the serosa and perforate into the mesentery, finally resulting in a confined abscess or an intestinal fistula after the lesions subsequently infiltrate adjacent intestinal walls.^[10]^ During this period, the patients are unlikely to present symptoms that are typical of fistula formation and, thus, are not diagnosed. The most common presenting symptoms of the primary small intestinal lymphoma are abdominal pain (84%), weight loss (81%), and diarrhea (39%), which are symptoms that also resemble other GI diseases. Only 23% of patients present with features of small intestinal perforation.^[11]^ Similarly, in the 2 cases mentioned above, abdominal pain and diarrhea were the 2 primary symptoms of the patients. The possible reasons why their perforation failed to be recognized are the poor general condition or that the lesion was confined. However, factors that may suggest the possibility of perforation include sudden increased pain or hematochezia. Patients with intestinal internal fistula will present with obstinate abdominal pain, intractable diarrhea, and malnutrition. In our case, the persistent diarrhea might be a result of colonization of the small intestine with colonic flora from the sigmoid colon.^[12]^ It is possible that the fistula between the small and large intestine offers a shortcut, causing intestinal content to fail to be well digested and absorbed, similar to short bowel syndrome.

CT and MRI are able to provide valuable information for staging the malignancy as well as assisting in preoperative planning.^[13]^ Certain imaging findings, such as a bulky mass with small intestinal loops wrapped around it, an air-contrast level and a fistulous tract with the tumor, may strongly suggest the formation of an intestinal fistula.^[5]^ A CT scan can delineate abnormally enhanced thickened intestinal wall and detect the site, shape and tract of the fistula. Because of its advantage allowing multiple sections to be observed, transabdominal US is more sensitive than CT for demonstrating the tract where the fistula is encountered; not only does US show the hypoechoic, thickened and rich-blood-flow intestinal wall, but it also enables the observation of dynamic movements of stiff and narrow lumen. US, CT, and magnetic resonance imaging (MRI) have high sensitivity and specificity for the diagnosis of intraabdominal fistulas, with similar diagnostic accuracies.^[14]^ Concerning the tumor bowel, differential diagnoses of a gas-forming infection or tumor necrosis are important if the imaging tests reveal the presence of gas alone in the tumor.^[5]^ A tumor-bowel fistula could be diagnosed if the tract between the involved bowels is detected or if there is air inside the lesions.

PET/CT is widely applicable for both the localization and viability of malignancies^[15]^; it is important in assessing disease activities, including both inflammation and necrosis.^[16]^ We can take full advantage of PET/CT to evaluate the prognosis and curative effect of patients after chemotherapy. For such patients, as observed in case 1, aiming at the targeted involvement site, the combination of PET/CT before chemotherapy and contrast-enhanced CT plays a vital role in the estimation of surgical indication and the optimal timing of surgery.^[16]^ However, the efficacy of PET/CT for evaluating the intestinal fistulization caused by lymphoma partially depends on the CT protocol. As in our 2 cases, PET/CT could not directly detect the fistula without the use of bowel contrast in the protocol.

Enteroscopy permits direct visualization of the mass and contrast of the mucosa of both intestinal segments.^[12]^ However, compared to other noninvasive imaging examinations, the enteroscope sometimes fails to reach the fistula because of the narrow and inflamed intestinal lumen. Biopsy under enteroscopy often yields a negative result because of deep lymphomatous lesions that increase the risks of bleeding and perforation. As in the first case presented here, intestinal lymphomas often do not receive a definitive diagnosis until the pathological results are obtained after surgery.

The prognosis for such cases is very poor; the survival rates associated with perforation at 6 months and 1 year was reported as 27.5% and 0%, respectively. Poor prognosis is attributing to several factors, which including the fast-growing T-cell lymphoma and resistance to chemotherapy regimens.^[17]^ Chemotherapy is a good option for patients with advanced intestinal lymphoma. Since data about incidents of postoperative complications are limited, the true value of selective operation for prognosis cannot be assessed.^[18]^ The 2 patients in this report tolerated the undesirable effects of chemotherapy well, and their conditions significantly improved in the follow-up period.

## 4 Conclusion

Intestinal fistula is a relatively rare complication of primary intestinal lymphoma, especially in patients who do not receive chemotherapy. Cross-sectional imaging modalities, such as US and CT, play an essential role in intestinal lymphoma fistula diagnosis as well as in treatment follow up.

## Author contributions

**Methodology:** Miaoqian Wang, Qian Yang, Wei Liu.

**Resources:** Wenbo Li, Hong Yang, Weixun Zhou.

**Software:** Ji Li.

**Writing – original draft:** Nan Zhuang, Qingli Zhu.

**Writing – review & editing:** Qingli Zhu.
